# *Marinobacter hydrocarbonoclasticus* NY-4, a novel denitrifying, moderately halophilic marine bacterium

**DOI:** 10.1186/2193-1801-2-346

**Published:** 2013-07-27

**Authors:** Rongpeng Li, Xiaoli Zi, Xinfeng Wang, Xia Zhang, Haofeng Gao, Nan Hu

**Affiliations:** College of Biotechnology and Pharmaceutical Engineering, Nanjing University of Technology, Nanjing, 211800 People’s Republic of China; Jiangsu Key Laboratory for Biomass-based Energy and Enzyme Technology, Huaian, 223300 People’s Republic of China

**Keywords:** 16S rRNA sequence, Denitrification, *Marinobacter hydrocarbonoclasticus* NY-4, Moderate halophile, Wastewater

## Abstract

The isolation and characterization of a novel halophilic denitrifying marine bacterium is described. The halophilic bacterium, designated as NY-4, was isolated from soil in Yancheng City, China, and identified as *Marinobacter hydrocarbonoclasticus* by 16S rRNA gene sequence phylogenetic analysis. This organism can grow in NaCl concentrations ranging from 20 to 120 g/L. Optimum growth occurs at 80 g/L NaCl and pH 8.0. The organism can grow on a broad range of carbon sources and demonstrated efficient denitrifying ability (94.2% of nitrate removal and 80.9% of total nitrogen removal in 48 h). During denitrification by NY-4, no NO_2_^-^-N was accumulated, N_2_ was the only gaseous product and no harmful N_2_O was produced. Because of its rapid denitrification ability, broad carbon use range and ability to grow under high salinity and pH conditions, NY-4 holds promise for the treatment of saline waste waters.

**Electronic supplementary material:**

The online version of this article (doi:10.1186/2193-1801-2-346) contains supplementary material, which is available to authorized users.

## Background

Excessive nitrate contamination in surface and ground water leads to many health and other problems, so that nitrogen (N) removal from water is of great importance (Paerl et al., 2002; Spalding and Exner, 1993). Denitrification is a bacterial process that can remove nitrogen from wastewater through the heterotrophic conversion of nitrate to N_2_ (Khardnavis et al., 2007)_._ This biological process is highly efficient at nitrogen removal, and does not produce any secondary pollution or residues (Song et al., 2011).

Denitrification occurs in many species of the bacteria and archaea (Zumft 1992), such as *Pseudomonas stutzeri* (Su et al., 2001), *Paracoccus denitrificans* (Robertson and Kuenen, 1983), *Alcaligenes faecalis* (Joo et al., 2005), *Bacillus* spp. (Kim et al., 2005), and *Microvirgula aerodenitrificans* (Patureau et al., 2001). Most denitrifying organisms used for biotechnological applications are mesophilic denitrifying bacteria (pH optima near 7.0 and with low or no salt tolerance). These strains are not always suitable for nitrate removal at high salt concentrations and high pH of industrial wastewaters (van der Hoek et al., 1987). Unlike domestic or landscape wastewaters, industrial wastewaters are complex matrices, which may contain many cell growth inhibitors (Hockenbury and Grady, 1977; Grunditz et al., 1998), such as high salt concentrations. As previously reported, when the salt concentration was higher than 6 g/L, a significant drop in the denitrification efficiency was observed, because many bacteria died when the salt concentration was higher than 6 g/L (Vendramel et al., 2011).

Use of bacterial strains that have both efficient denitrifying ability and salt tolerance in industrial wastewater treatment may solve this problem. As reported, denitrification under moderately halophilic conditions (pH range from 7.0 to 9.0 and a salt concentration under 2M Na^+^) has been shown for several members of the *Gammaproteobacteria*, such as *Thioalkalivibrio spp.* (Sorokin et al., 2001; Sorokin and Kuenen 2005) and *Halomonas spp.* (Berendes et al., 1996; Mormille et al., 1999; Peyton et al., 2001; Romano et al., 2005; Boltyyansakaya et al., 2004, Boltyanskaya et al. 2007). Some *Halomonas* strains were also demonstrated to actively denitrify under highly halophilic conditions (pH 9.0 and a salt concentration of 4M Na^+^) (Klup et al., 2007; Shapovalova et al., 2008). It is unfortunate that all these reported halophilic species only grew and actively denitrified under aerobic conditions. Because of the low concentrations of dissolved oxygen in industrial wastewaters, these reported aerobic halophilic species were not suitable for application in treatment of industrial wastewater (Shapovalova et al., 2008).

The objective of this research was to isolate and characterize new halophilic denitrifying bacteria with better potential for saline industrial wastewater treatment. To achieve this objective, sediment samples were collected from Xintan Saltern, Yancheng City, Jiangsu Province, China. A halophilic bacterium NY-4 was isolated and identified as *Marinobacter sp*. by 16S rRNA analysis. NY-4 has a high anaerobic denitrifying efficiency (94.2% nitrite removal and 80.9% total nitrogen removal in 48 h), moderate salt tolerance (from 20 g/L to 120 g/L), pH tolerance (from 7.0 to 9.0) and broad carbon use range, which makes NY-4 a promising inoculant for saline industrial wastewater treatment.

## Materials and methods

### Strains, medium and culture condition

NY-4 was isolated from soil in Xintan Saltern, Yancheng City, Jiangsu Province, China, and maintained in 8% glycerol HSLB plus medium (High Salinity LB medium, LB with 80 g/L NaCl) at −80°C. For the isolation of bacterial strains, a soil sample was suspended and vortexed thoroughly in sterile H_2_O and serially diluted 10-fold four times using sterile H_2_O. One hundred μl of the 10^-4^ dilution was spread on an HSLB plus plate and incubated at 30°C for 48 h. Single colonies were selected. NY-4 was obtained after strict investigation of its purity.

The following indicates the contents of each medium that was prepared: Denitrification medium (DM, g/L): NaNO_3_, 0.607; KH_2_PO_4_, 5; NaCl, 80, trace element solution, 1 ml, pH, 7.0-8.0; the amount of carbon source varied by experiments; trace element solution (g/L): Na_2_EDTA, 63.7; ZnSO_4_, 2.2; CaCl_2_, 5.5; MnCl_2_^.^4H_2_O, 5.06; FeSO_4_.7H_2_O, 5.0; Na_2_MoO_4_^.^4H_2_O, 1.1; CuSO_4_^.^5H_2_O, 1.57; CoCl_2_^.^6H_2_O, 1.61, pH, 7.0-7.5. High Salinity LB plus medium (HSLB, g/L): tryptone, 10; yeast extract, 5; NaCl, 80; KCl, 5; MgSO_4_^.^7H_2_O, 2.5; pH, 7.0-7.5. To prepare HSLB plus medium plates, 2% (w/v) agar was added. For strain maintenance, glycerol was added into LB plus liquid medium to a final concentration of 8% (v/v).

NY-4 was transferred from an HSLB plus frozen culture maintained at −80°C to a fresh HSLB plus medium plate and incubated at 30°C for 24 h. NY-4 from the plate was inoculated into the HSLB plus liquid media and incubated at 30°C at 150 rpm for 18 h (about OD_600_ at 5.0) as the preculture. The preculture was inoculated into DM medium and cultivated as described for each experiment.

### Electron microscopy observation

Detailed cell shapes and flagella were observed both by scanning electron microscopy and transmission electron microscopy. For scanning electron microscopy analysis, bacteria were fixed with 1% glutaraldehyde overnight and then dehydrated in ethanol completely. Cells were coated with gold-palladium and observed with a JSM-5610LV scanning electron microscope (JEOL Ltd., Tokyo, Japan) (Bertrand et al., 1990). For transmission electron microscopy examinations, cells were negatively stained with phosphotungstic acid according to the method of Jahn (1986) and observed with an H-7650 transmission electron microscope (Hitachi, Tokyo, Japan).

### 16S rRNA analysis

The 16S rRNA was amplified by PCR using the universal bacterial primers 27F (5’-AGAGTTTGATCCTGGCTCAG-3’) and 1492R (5’-GGYTACCTTGTTACGACTT-3’) (Lane 1991) and was sequenced by TAKARA Corp. (Dalian, China). The sequence was submitted to a Blast search to compare with available 16S rRNA gene sequences in GenBank of the NCBI database (Altschul et al., 1990). A neighbor-joining tree was constructed using the MEGA 4.0 program (Kumar 2004).

### Denitrifying assay

A 1.5 mL of preculture of NY-4 in HSLB plus medium was inoculated in 150 mL of DM liquid medium sealed with 15 mL paraffin wax, contained in 250-mL flasks. The flasks were then incubated at 30°C in a constant temperature incubator. The cultures were sampled periodically to determine bacterial growth and the concentration of NO_2_^-^-N, NO_3_^-^-N and the total nitrogen (TN) were measured every 12 h during incubation for 72 h. The same denitrification process was performed to detect gaseous nitrogen products, with the exception that tightly sealed infusion bottles (150 mL DM/250-mL flask) were used. N_2_ or N_2_O product was monitored in DM medium at the end of cultivation (72 h).

### Effect of culture conditions on denitrification performance of NY-4

The effects of the NaCl concentration, carbon source, C/N ratio, and initial pH on the denitrification performance of NY-4 were investigated by single factor tests (Tang and Luo, 2008).

In experiments to determine the role of the carbon source, sodium succinate, trisodium citrate, sodium acetate and ethanol were used as the sole carbon source in DM. The other conditions were as follows: initial concentration, 100 mg/L of NO_3_^-^-N; C/N = 10; 80 g/L of NaCl; initial pH of 8.0; and temperature of 30°C. In the experiments to test the effect of C/N ratio, trisodium citrate served as the sole carbon source, and the concentration was varied to yield different C/N ratios (1, 5, 10, 15, 20, 25 and 30) at a fixed concentration of NO_3_^-^-N (100 mg/L). The other conditions were the same as those used for the carbon source experiments. To observe the effects of salinity and initial pH on nitrogen removal, the concentration of NaCl in DM was varied from 0 to 160 g/L (at intervals of 20 g/L), and the initial pH from 6.0 to 10.0 (at intervals of 1.0 pH units). These tests were conducted at a C/N ratio that demonstrated optimum growth conditions. Samples were collected for the determination of bacterial growth rate, NO_2_^-^-N, NO_3_^-^-N and TN during incubation for 48 h.

### Analysis methods

Bacterial cells were stained with a Gram staining method (Magee et al., 1975), observed and counted in blood counting chamber, using an optical microscope (LW40B, Cewei Corp. Ltd., China). Growth rate was calculated as change in the number of incerased cells vs culturing time during the exponential phase of growth.

Nitrate (NO_3_^-^-N), nitrite (NO_2_^-^-N), and total nitrogen (TN) were determined according to standard methods (APHA et al., 1998), including diphenylamine spectrophotometry, *N*-(1-naphthyl)-1, 2-diaminoethane dihydrochloride spectrophotometry, and potassium persulfate digestion UV spectrophotometric methods, respectively.

The gaseous nitrogen products N_2_O and N_2_ from headspace were analyzed by gas chromatography (GC9790-2, Guji Corp. Ltd., China). The conditions were as follows: Packed GC Column [Porapak Q] (mesh size 60/80, Φ3×3 m, Agilent); sample injection port and detector temperature set at 70°C and 100°C, respectively. Gas flow: carrier H_2_ 1.2 mL/min; the added quantity was 600 μL.

### Amplification of the nitrite reductase gene and nitrous oxide reductase of NY-4

Fragments of the *nir*S gene of NY-4 were amplified using the primers cd3F and cd4R, developed by Michotey et al. (2000). The primer sequences were cd3F: 5’- GT(A/T/C/G)AA(T/C)GT(A/T/C/G)AA(A/G)GA(A/G)AC(A/T/C/G)GG-3’, cd4R: 5’-AC(A/G)TT(A/G)AA(T/C)TT(A/T/C/G)CC(A/T/C/G)GT(A/T/C/G)GG-3’. Fragments of the *nir*K gene of NY-4 were amplified using primers F1aCu and R3Cu, developed by Hallin et al. (1999). The primer sequences were F1aCu: 5’-ATCATGGT(C/G)CTGCCGCG-3’, R3Cu: 5’-GCCTCGATCAG(A/G)TTGTGGTT-3’. Fragments of the *nos*Z of NY-4 were amplified using primers nosz47F and nosz1776R, referenced with the reported potential *nos*Z gene (GenBank, GI: 107515115). The primer sequences were nosz47F: 5’-GAGGTTTCCGAAGGTGGTCTG-3’, nosz1776R: 5’-GGTCACGGAAGCGGTCTGC-3’. The PCR conditions used were according to the references described for each primer pair. Aliquots of 10 μl of the reactions were analyzed by electrophoresis on 2% (w/v) agarose gels. Bands were visualized by UV excitation and sequenced by TAKARA Corp. (Dalian, China). The sequences were compared with available reported gene sequences in GenBank using the BLAST program.

### Statistical analysis

All results were shown as the average of at least three independent experiments; variation was expressed as SD. Student’s *t-test*, analysis of variance (ANOVA) and the Duncan’s multiple range tests were used to determine whether the nitrite-N degradation rate differed significantly between treatments (P < 0.05). All statistics were performed using SPSS for Windows version 11.0.

## Results

### Identification of NY-4

**Figure 1:**
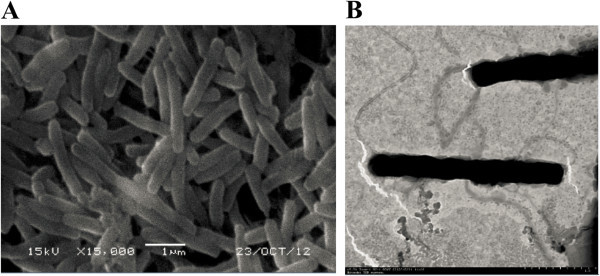
**Scanning (A) and transmission (negative staining, B) electron micrographs of** ***M. hydrocarbonoclasticus*** **NY-4.**

NY-4 was heterotrophic and able to grow under both aerobic and anaerobic conditions. The colonies of NY-4 were white, circular in shape, with semitransparent, slabby, wet and smooth surfaces on LB plus plates. The cells were Gram-negative, bacilliform with a size of 0.2 - 0.3 μm in diameter and 2.0 - 3.0 μm in length (Figure 1A), and were motile with general flagellum (Figure 1B). Almost the entire 16S rRNA gene (1502 nt) was PCR-amplified and sequenced, and the sequence was submitted to GenBank under the accession number JN903898. The BLAST results indicated that NY-4 was closely related to members of genus *Marinobacter*, showing the highest 16S rRNA gene sequence similarity (99%) to *Marinobacter hydrocarbonoclasticus*. The phylogenetic analysis also showed that NY-4 was grouped together with *M. hydrocarbonoclasticus* in the tree with 100% bootstrap support (Additional file 1: Figure S1).

**Figure 2:**
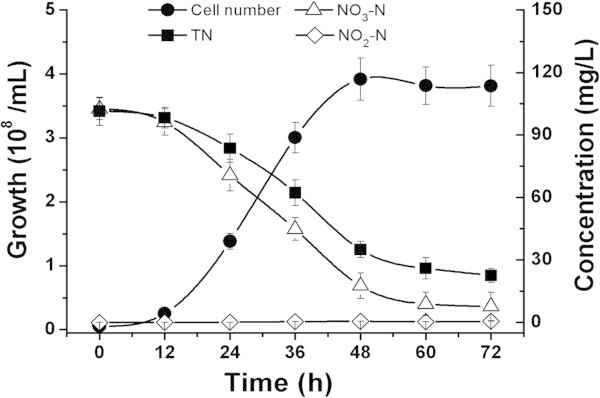
**Time course of** ***M. hydrocarbonoclasticus*** **NY-4 denitrification in anaerobic.**

We assessed the anaerobic denitrification performance of NY-4. When inoculated in denitrification DM medium, NY-4 grew quickly, concurrent with a significant and rapid consumption of NO_3_^-^-N (about 85% in 48 h), showing a high efficiency in denitrification and nitrogen removal capacity (Figure 2). Surprisingly, no NO_2_^-^-N accumulated was detected throughout the incubation period, which meant that NY-4 could rapidly use NO_2_^-^-N as an electron acceptor reduced into gaseous nitrogen products (Shao and Yu, 2008).

### Factors affecting denitrification of NY-4

We tested the effects of the NaCl concentration, carbon source, C/N ratio, and initial pH on the denitrification performance of NY-4 by single factor tests (Tang and Luo, 2008). In most different cultivation conditions, NY-4 had grown at a maximum cell density and entered the stationary phase when the cultures were sampled at 48 h, except for no growth in extreme unsuitable conditions. In addition, cells of NY-4 at the similar time is in the same growth phase so that the data of nitrogen removal were significative and comparable with each other.

#### Salinity concentration

As the main aim in this study was to isolate and identify strains with both effective denitrifying ability and high tolerance to salinity, we first assessed the denitrifying ability of NY-4 cultured in DM medium with a large range of salt concentrations, from 0 to 160 g/L NaCl.

**Figure 3:**
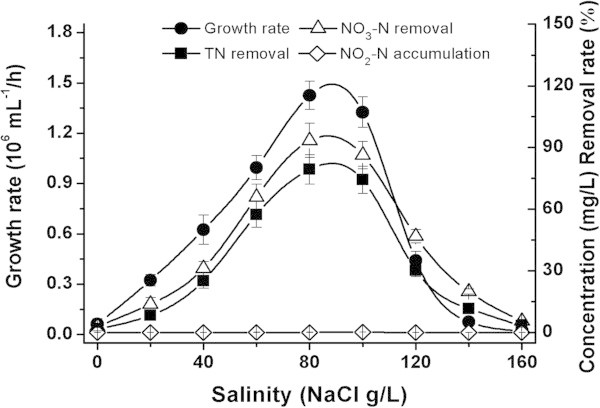
**Effects of salinity on the denitrification of** ***M. hydrocarbonoclasticus*** **NY-4.**

As shown in Figure 3, NY-4 could not grow in DM medium without NaCl. The anaerobic growth rate, NO_3_^-^-N and TN removal rates of NY-4 increased together until the NaCl concentration gradually reached 80 g/L and decreased with the continuous increase in NaCl concentration above 80 g/L. With this concentration, the growth rate of NY-4 was 1.43 × 10^6^ mL^-1^/h and removal rate of NO_3_^-^-N and TN were 93.4% and 79.3% respectively.

#### Carbon source

The carbon source is one of the most important factors affecting denitrifying ability. During the denitrification process, cells use the carbon source as an electron donor and gradually reduce nitrate to N_2_, thus removing organic matter and nitrate simultaneously. Her and Huang (1995) proposed that the structure and molecular content of the carbon source were significant factors affecting the efficiency of denitrification.

We tested a range of carbon sources to determine the best carbon source affecting the denitrification rate of NY-4 and found it was sodium succinate. When grown on sodium succinate, NY-4 grew the best and exhibited the most efficient denitrifying ability, with a growth rate of 1.38 × 10^6^ mL^-1^/h, while the NO_3_^-^-N and TN removal percentages reaching 92.9% and 83.1% respectively (Table 1). The results in Table 1 also showed that NY-4 did not grow very well and that denitrification was less efficient with other carbon sources.

**Table 1:** **Effect of carbon source on the denitrifying performance of NY-4**

Carbon source	Growth rate (10^6^mL^-1^/h)	NO_3_^-^-N removal (% 48 h)	TN removal (% 48 h)
Sodium succinate	1.29 ± 0.13	88.8 ± 2.1	71.0 ± 3.2
Trisodium citrate	1.38 ± 0.18	92.9 ± 3.5	83.1 ± 2.4
Sodium acetate	0.68 ± 0.09	52.1 ± 3.3	43.9 ± 4.1
Ethanol	0.10 ± 0.01	8.4 ± 1.6	6.8 ± 1.2

Generally speaking, a carbon source with a simple, small molecular structure would be more favorable for denitrification. For NY-4, sodium succinate was the best carbon source, probably because sodium succinate can easily enter the tricarboxylic acid cycle (TCA) and rapidly provide energy and reduction force (Zheng et al., 2012).

#### C/N ratio

**Figure 4:**
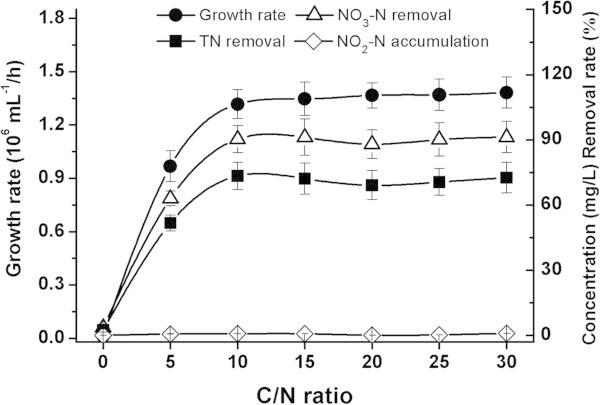
**Effects of C/N ration on the denitrification of** ***M. hydrocarbonoclasticus*** **NY-4.**

The effect of altering the C/N ratio is shown in Figure 4. When the C/N ratio was low, such as 1:1, NY-4 did not grow and nearly no NO_3_^-^-N and no TN were removed. Similar to other denitrifying strains (Lesley et al., 1983), an increase in organic carbon concentration yielded an increase in the denitrification efficiency of anaerobic denitrifier NY-4. When the C/N ratio was 10:1, the growth rate of NY-4 (1.31 × 10^6^ mL^-1^/h) and removal of both NO_3_^-^-N (90.3%) and TN (73.3%) reached its peak. However, when the C/N ratio was higher than 10:1, there was no further improvement in the bacterial growth and nitrogen removal. Taking cost effectiveness into consideration, a C/N ratio of 10 was selected as the optimum operating condition for subsequent assays.

#### pH

**Figure 5:**
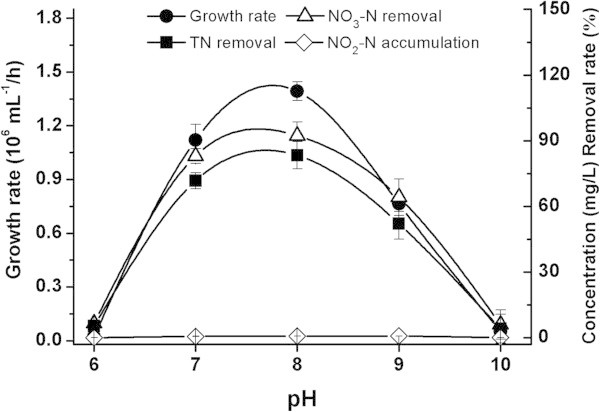
**Effects of pH on the denitrification of** ***M. hydrocarbonoclasticus*** **NY-4.**

The effects of initial pH are shown in Figure 5. NY-4 could not grow under highly acidic (pH < 6.0) or highly alkaline (pH > 10.0) conditions. Under mesophilic conditions (pH 6.0 - 8.0), cell growth and TN removal increased quickly with maximal cell growth rate (1.39 × 10^6^ mL^-1^/h), NO_3_^-^-N (92.3%) and TN removal (83.4%) at pH 8.0. In the pH range from 8.0 to 10.0, cell growth rate and nitrogen removal dropped quickly while the denitrifying ability was lost completely in pH 10.0. For practical wastewater treatment applications, a pH of 8.0 was chosen as the optimum pH for the anaerobic denitrification performance for NY-4.

Interestingly, even with all the different factors we changed and assessed, no obvious NO_2_^-^-N accumulation was detected, which meant that any NO_2_^-^-N produced was quickly used as an electron donor and converted to N_2_O by NY-4.

### Detection of gaseous products of NY-4

At the end of the incubation of NY-4, lots of bubbles were observed in the cell cultures. Because no NO_2_^-^-N accumulation was detected, we presumed that any NO_2_^-^-N formed by denitrification was quickly converted to gaseous products. The gaseous nitrogen products of denitrification are N_2_O or N_2_ (Jones et al., 2011), which has been demonstrated in some species of bacteria such as *Alcaligenes faecalis* (Joo et al., 2007). Nitrous oxide and N_2_ from the culture headspace were analyzed by gas chromatography. As shown in Additional file 2: Figure S2, the peak of a standard sample of N_2_ emerged between 0.664 min and 1.013 min, while the peak of a standard sample of N_2_O emerged between 2.063 min and 3.026 min. The peak of gaseous nitrogen production from NY-4 appeared between 0.673 min to 1.002 min, corresponding to the standard N_2_ peak. A small peak was detected at 2.095 min, corresponding to N_2_O. This result demonstrated that NY-4 could produce both N_2_O and N_2_ through its denitrification process, but N_2_O was rapidly converted to N_2_ so that negligible N_2_O was detected (Additional file 2: Figure S2).

### Amplification of denitrification nitrite reductase and nitrous oxide reductase of NY-4

Denitrification is part of the bioenergetic ability of the bacterial cell, where the nitrate and nitrite and the gaseous NO and N_2_O serve instead of dioxygen (O_2_) as terminal acceptors for electron transport phosphorylation. It is clearly the role of denitrification in the global N cycle and in cellular bioenergetics that makes a detailed knowledge of this process essential (Zumfit 1997).

Denitrification will be considered as the assemblage of nitrate respiration, nitrite respiration combined with NO reduction, and N_2_O respiration:

NO_3_^-^→NO_2_^-^→NO→N_2_O→N_2_

**Figure 6:**
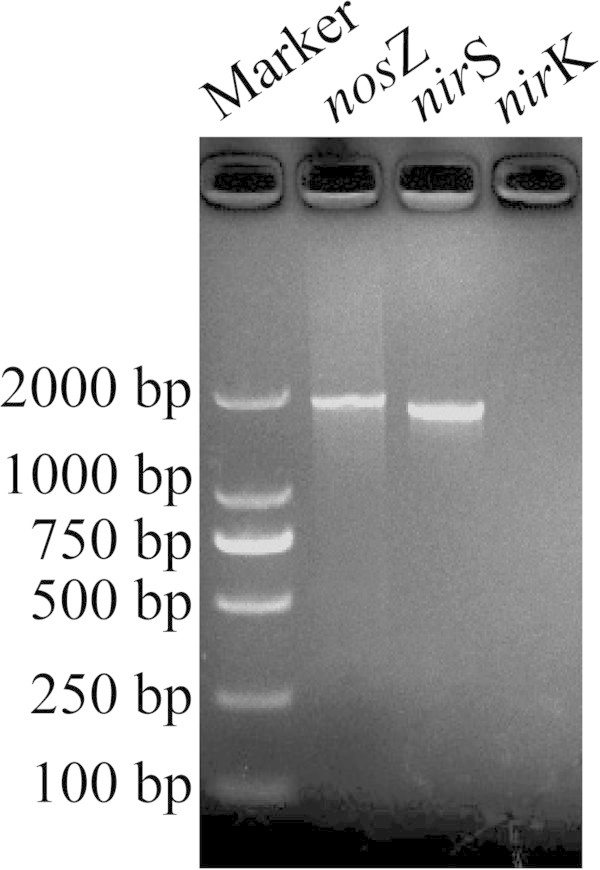
**Amplification profiles of** ***nir*** **S,** ***nir*** **K and** ***nos*** **Z gene from** ***M. hydrocarbonoclasticus*** **NY-4.**

Nitrite reductase functional genes encode the enzymes that play a key part in the bacterial denitrification process (Song et al., 2011). There have been many reports on the composition and gene sequence of the nitrite reductase (*nir*) gene, and there are two types of *nir* gene, *nir*S (cytochrome cd1-type enzyme) and *nir*K (copper-based enzyme) (Brake et al., 2000; Ole et al., 2009; Stouthamer 1992). The results of PCR amplification of nitrite reductase genes *nir*S and *nir*K from NY-4 are shown in Figure 6. The *nir*S (1725 nt) amplification was positive, while the *nir*K amplification was negative. The BLAST result showed this *nir*S gene shared 97% homology with reported *M. hydrocarbonoclasticus* potential *nir*S gene (GenBank, GI: 4657102). These results indicated that the NY-4 had the *nir*S form of the nitrite reductase gene.

In the denitrifying process, three gases (NO, N_2_O and N_2_) can be produced. Because of the high activity and specificity of NO reductase, NO is hardly accumulated, so the main gaseous products of the denitrifying process are N_2_O and N_2_. N_2_O is an important greenhouse gas, with ~320-fold higher impact than CO_2_ on greenhouse effect (Lashof and Ahuja, 1990). For this reason it was important to isolate strains that completely denitrify NO_3_^-^-N to N_2_. The presence of the *nosZ* gene, encoding nitrous oxide reductase, indicates whether the strain has the genetic capacity for complete reduction to N_2_ (Jones et al., 2011).

We analyzed the gases produced by NY-4 and found that the majority of gas produced was N_2_ with very little N_2_O accumulation. We were able to amplify the *nos*Z gene of NY-4 (1896 nt) (Figure 6). The BLAST result showed this *nos*Z gene shared 94% homology with a reported *M. hydrocarbonoclasticus* potential *nos*Z gene (GenBank, GI: 107515115). We postulate that the nitrous oxide reductase encoded by the *nosZ* gene of NY-4 had a very high enzymatic activity so that the N_2_O produced was quickly converted to N_2_.

## Discussion

As we know, industrial wastewaters contain a large amount of nitrates and salt (Glass and Silverstein, 1997; Hirata et al., 2001; Labbe et al., 2003). van der Hoek et al. (1987) and Clifford and Liu (1993) showed that denitrification was possible under 3% (w/w) NaCl conditions. However, the denitrification rate of saline wastewaters decreased under highly saline conditions (Yang et al., 1995). Therefore, nitrogen removal from saline wastewater has been considered to be difficult (Yoshie et al., 2004). The discovery of denitrifying halophilic bacteria was thought to solve this problem and many such strains have been reported. Most of these strains only grow under aerobic conditions, which limits their use in treatment of low DO industrial wastewaters.

In this study, a novel *M. hydrocarbonoclasticus* NY-4 was shown to almost completely reduce nitrate under optimum conditions (80 g/L NaCl and pH 8.0). Compared with other halophilic species, NY-4 could grow faster (maximum at 1.39 × 10^6^ mL^-1^/h) and to a higher density (3.92 × 10^8^ mL^-1^) under anaerobic conditions. *M. hydrocarbonoclasticus* NY-4 also rapidly converted NO_3_^-^-N into N_2_, without NO_2_^-^-N or N_2_O accumulation. Generally, NY-4 could reduce nitrate within a NaCl concentration range from 20 to 120 g/L and pH range from 7.0 to 9.0. The strain was also able to use a range of carbon sources for growth and denitrification, such as sodium succinate, trisodium citrate and sodium acetate. Because of its denitrification ability, broad carbon use range and high tolerance to salinity and pH, NY-4 holds promise for the treatment of saline wastewaters.

We also tested the denitrifying ability of NY-4 under aerobic conditions (data not shown). The best TN removal was only about 40% under aerobic conditions, which is no better than other reported strains in nitrogen removal. Considering the low dissolved oxygen in industrial wastewaters, NY-4 remains a promising potential inoculant for saline wastewater treatment.

## Electronic supplementary material

Additional file 1: Figure S1: Phylogenetic tree generated from an alignment of the 16S rRNA. (TIFF 768 KB)

Additional file 2: Figure S2: GC profiles of the standard gas samples and gaseous production of *M. hydrocarbonoclasticus* NY-4. (TIFF 492 KB)
