# Supplementary figures and images for: Marinobacter hydrocarbonoclasticus NY-4, a novel denitrifying, moderately halophilic marine bacterium
Source: Springerplus. 2013 Jul 27;2:346. doi: 10.1186/2193-1801-2-346 (PMC4269976; doi:10.1186/2193-1801-2-346)

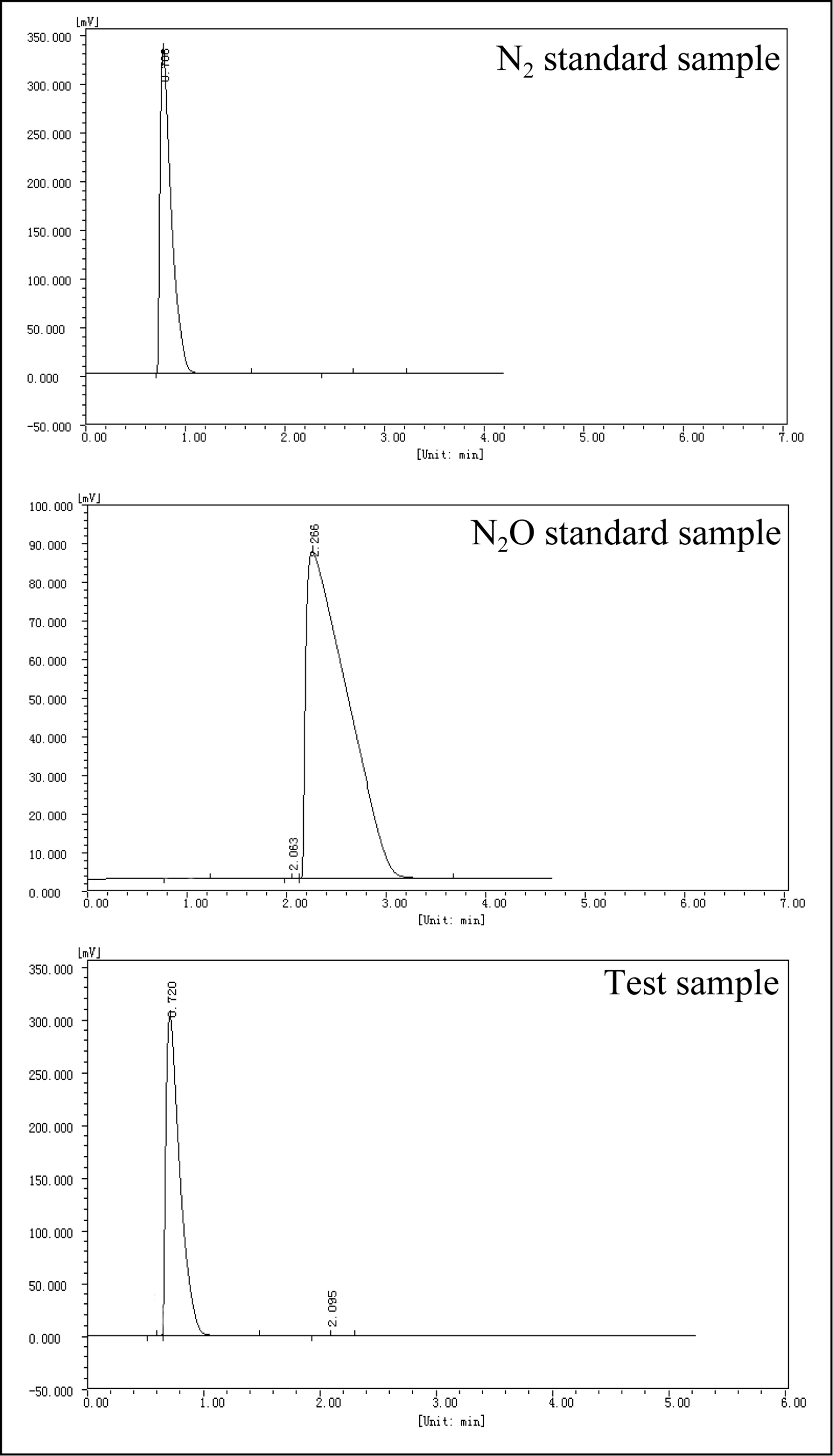

Supplement: Supplementary file 2 — Additional file 2: Figure S2: GC profiles of the standard gas samples and gaseous production of M. hydrocarbonoclasticus NY-4. (TIFF 492 KB) [file 40064_2013_409_MOESM2_ESM.tiff]
